# The correlation between optical coherence tomography retinal shape irregularity and axial length

**DOI:** 10.1371/journal.pone.0227207

**Published:** 2019-12-30

**Authors:** Stewart Lake, Murk Bottema, Keryn Williams, Karen Reynolds

**Affiliations:** 1 Ophthalmology, College of Medicine and Public Health, Flinders University, Adelaide, South Australia; 2 Medical Device Research Institute, College of Science and Engineering, Flinders University, Adelaide, South Australia; University of Copenhagen, DENMARK

## Abstract

**Purpose:**

To describe the retinal contour in optical coherence tomography (OCT) images, and report the relationship between retinal contour and axial length.

**Methods:**

Retinal contour was defined by the path of the retinal pigment epithelial (RPE) line in macular and extra-macular OCTs of 70 eyes of 70 participants recruited from ophthalmology clinics in South Australia. The shape of this contour was described by the best-fit curvature (K), and Fourier analysis of the difference between K and the RPE. The Fourier transformation was summarised by total difference (sumdiff), maximum single frequency difference (MaxE), and root mean square difference (rmse) between each B scan residual and the average normal. All-of-eye and regional median and interquartile range (IQR) shape features were correlated to axial length.

**Results:**

Retinal shape irregularity measured by Fourier transformation correlated with axial length: all-of-eye median and IQR sumdiff (***ρ*** = 0.66 and ***ρ*** = 0.60 respectively), median and IQR rmse (***ρ*** = 0.67 and ***ρ*** = 0.48), median MaxE (***ρ*** = 0.61), and IQR K (***ρ*** = 0.61) all correlated with axial length. Correlation with axial length was also seen in these parameters for 11 of 17 regions. Retinal irregularity was greatest at the macula and in inferior regions.

**Conclusion:**

Retinal OCT shape becomes increasingly irregular as axial length increases. The range of curvature correlates with axial length, while median curvature does not.

## Introduction

Optical coherence tomography (OCT) is an essential tool in current ophthalmic care. Information considered in retinal OCT images typically relates to structural features of and around the retina. Awareness of its reliability and usefulness, combined with the volume of information within OCT images has led to the development of multiple tools for quantitative analysis of retinal OCT scans to guide disease management.[1] Very little quantitative analysis has been undertaken on the shape of the retinal contour. Shape is known to affect retinal diseases including myopic traction maculopathy,[2] dome shaped maculopathy,[3] and degenerative myopic retinopathy.[4,5] Analyses of globe shape have been performed with magnetic resonance imaging (MRI) of the whole eye.[6–8] Myopic eyes are known to be more irregular in shape in magnetic resonance imaging globe reconstructions than non-myopic eyes, with staphyloma at the posterior pole a common feature of high myopia.[9]

OCT has been used to describe the shape of the macula region in high myopia.[10] Local variations in curvature have been described in eyes with and without staphyloma,[11] but the difference between best fit curvature and retinal contour has not been analysed. MRI can image the entire eye, but has limited resolution of 0.5 mm,[8] whereas OCT resolution approaches 2 μm in the axial direction, and up to 10 μm laterally. OCT is limited by the volume of retina that can be acquired in a single scan, although the volume captured in a single cube continues to increase with newer imaging devices.[12] Extra-macular OCT images have been used to describe features of the peripheral retina, both with isolated peripheral retinal images,[13,14] and with the merging of separate scans to create a longer optical section,[15] although analysis of the shape of extra-macular areas has not been reported. Alignment of adjacent B scans requires knowledge of image translation and rotation in three dimensions, which is difficult when the true three-dimensional shape of the image is unknown.[16]

**Fig 1:**
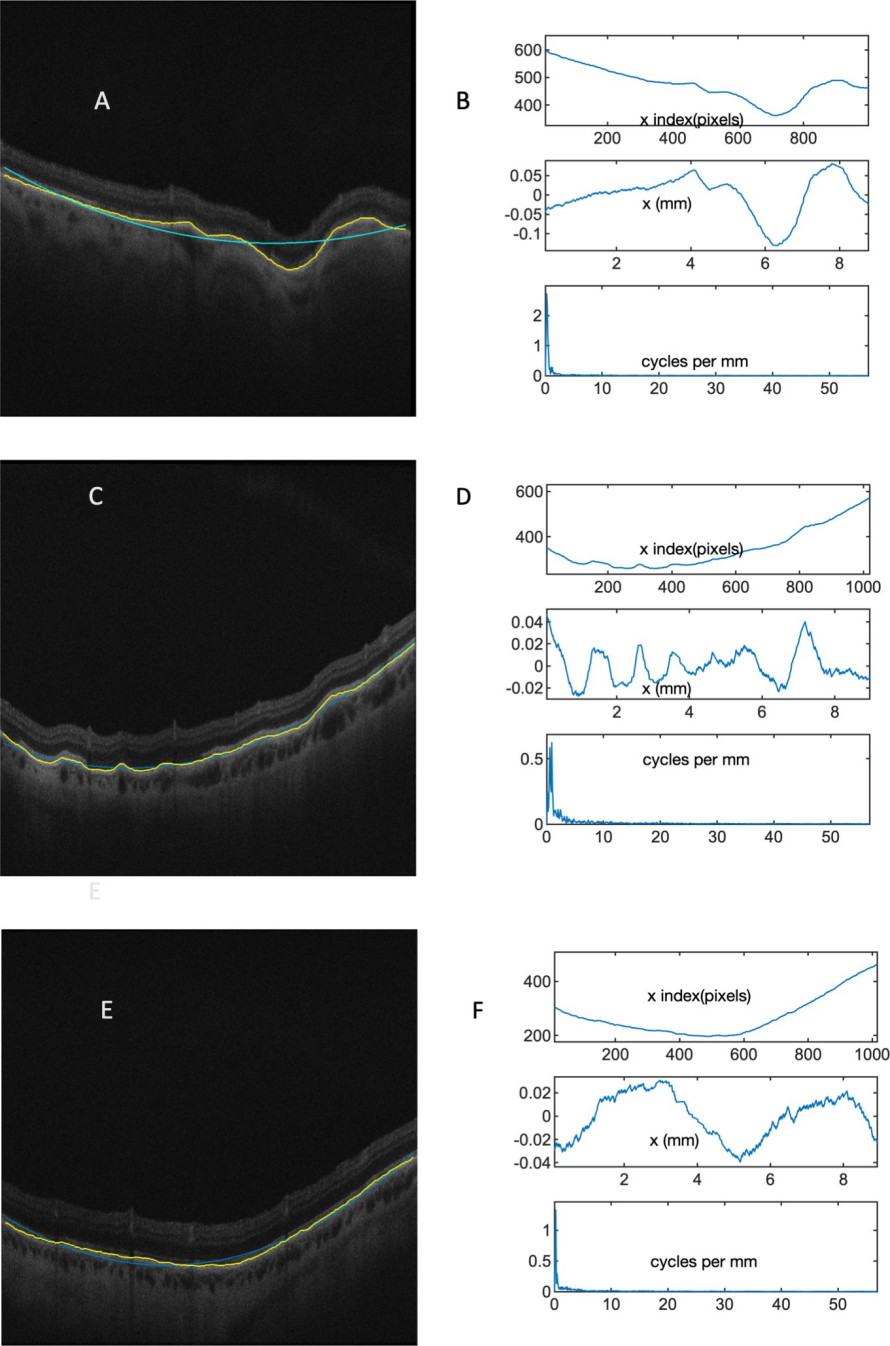
Retinal shape analysis. Retinal shape was taken from the contour of the retinal pigment epithelium in OCT B scans. Figs A, C, & E: the retinal contour is highlighted in yellow in the B scan images, and this contour is used for shape analysis. The quadratic best fit curve is superimposed in blue on the OCT images. Figs B, D, & F present the Fourier analysis of OCTs A, C, and E respectively. The B scan retinal contour is plotted in the uppermost panel of B, D, F. The middle panel in B, D, and F present the residual after the best-fit quadratic curve has been subtracted, with the Fourier transform of the residual in the lowermost. There is very little shape information at higher frequencies in the frequency domain.

This report describes the results of shape analysis of retinal OCT contour, using the orientation independent measures of curvature and Fourier analysis of the deviation of the retinal contour from that curvature (Fig 1). These features describe local shape abnormalities present within each B scan, and are correlated with the axial length of the eye, the primary determinant of myopia.[17] The retinal shape is represented by the contour or path that the retinal pigment epithelium line takes in B scans. The area of retina examined includes extra-macular regions. The novel shape analysis method described here requires only data easily available in the clinic, and has the potential to provide quick, high resolution, cost effective local shape information relevant to eye health.

## Methods

Seventy eyes of 70 participants were recruited from ophthalmology clinics in South Australia. Their reason for attending the clinic were new onset floaters (51 eyes, all with posterior vitreous detachment), optometric initiated review for retinal assessment of myopic eyes (16 eyes), ocular hypertension (one eye, no structural or posterior segment abnormality), and previous retinal detachment or retinal tear in the contralateral eye (two eyes, both had posterior vitreous detachment with no retinal pathology). Myopic maculopathy was present in 13 participants: 3 with a tessellated fundus (category 1 in the International photographic classification of myopic maculopathy[18]), 4 with diffuse chorio-retinal atrophy (category 2), and 6 with patchy choroidal atrophy (category 3). Participant age range was 33–83 years (median 62 years), with axial length from 21.11–36.88 mm (median 24.52 mm), measured with the Zeiss IOLMaster (Carl Zeiss Meditec AG, Germany). There were 40 right eyes, and 30 left eyes, and 37 were female, 33 male. The study was undertaken with Southern Adelaide Clinical Human Research Ethics Committee approval, in accordance with the Declaration of Helsinki. After written informed consent, eyes were dilated with one drop of tropicamide 1% (Bausch & Lomb, Chatswood, Australia), and one drop of phenylephrine 2.5% (Bausch & Lomb, Chatswood, Australia).

### Image acquisition

**Fig 2:**
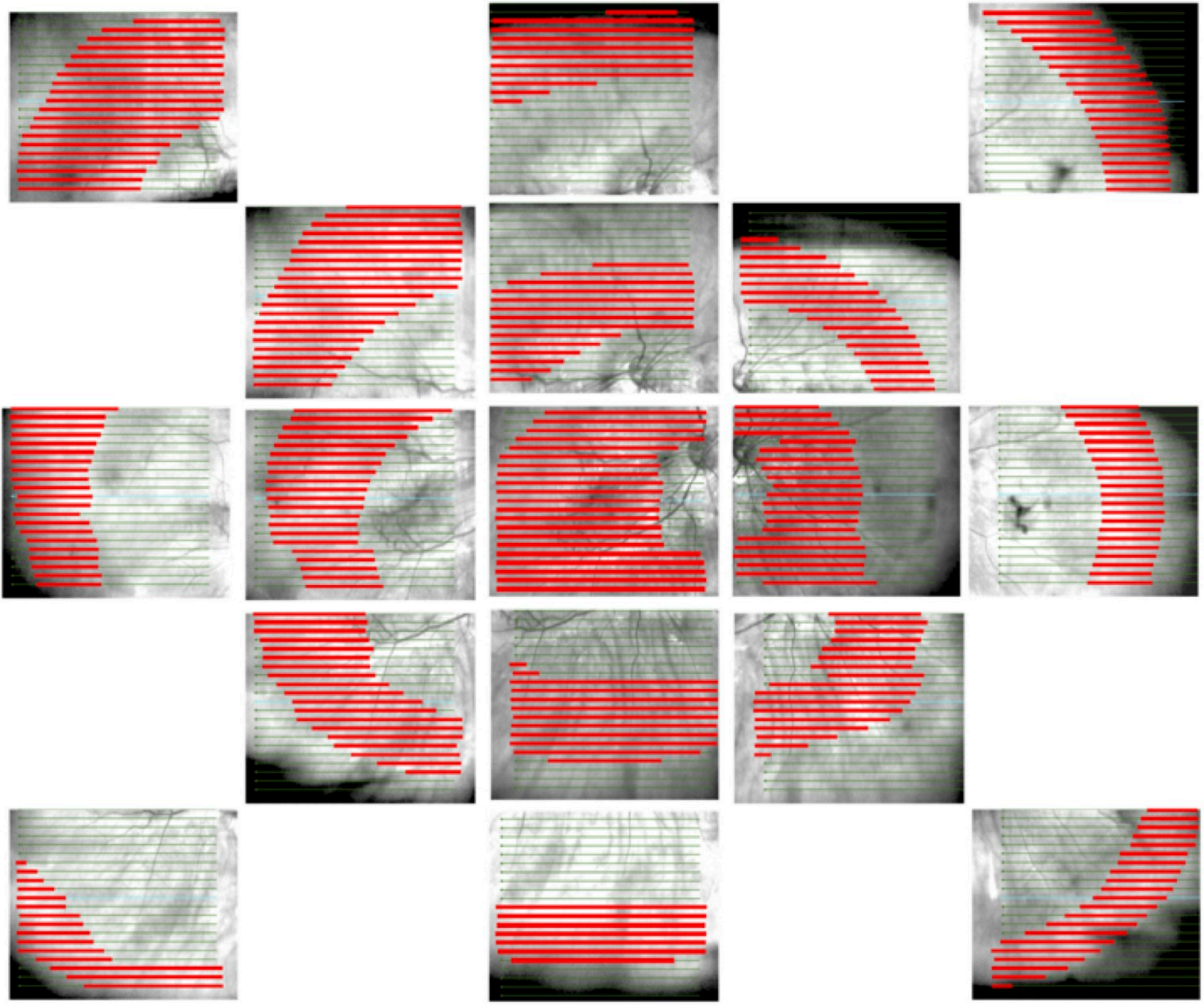
Example of retinal contour sampling. Exploded retinal map from a highly myopic eye (axial length 28.25 mm) illustrating areas imaged with the OCT. Each horizontal line marks the location of a B scan. The OCT cube samples a volume with a depth of 2 mm, and as the retina curves antero-posteriorly through the cube, is only seen in the section of each B scan highlighted in red. Fourier transform moduli were corrected to compensate for different retinal image lengths in different B scans.

Throughout this report the term B scan applies to a single OCT retinal image, and the term cube refers to a set of parallel B scans acquired together using a standard clinical protocol. The Zeiss Cirrus 5000 OCT (Carl Zeiss Meditec AG, Germany) was used for all images. Acquisition protocol for all scans was the HD21 cube, taking 9 mm horizontal scans spaced 0.4 mm apart. This is a multiple pass composite image, which eliminates within B scan axial artefacts arising from subject movement.[19] All scans were taken with a horizontal orientation for consistency within and between eyes and to maximise the sampled retinal area in each cube. Changing scan orientation from the horizontal would reduce the area imaged in a single cube and potentially cause variation in sampling between eyes. The first cube was taken with the participant looking directly toward the OCT fixation point (the macula cube). Subsequent OCT cubes were taken in 8 positions of gaze: superiorly, inferiorly, horizontally to the left and right, up and right, up and left, down and right, and down and left. At least 2 OCT cubes were taken in each direction of gaze, one at the edge of the macula cube, and the second more peripheral. Each of these locations was considered a “region” for image feature analysis. A set of scanning laser ophthalmoscope images from OCT cubes of one eye is shown in Fig 2. Due to the curvature of the eye, in some regions the retina passes obliquely through the rectangular B scan window, and the retinal image does not always cross the entire width of the B scan, nor does the retina appear in all B scans in a cube. Analysis was performed with the 25,870 B scans from 1,376 OCT cubes that contained retinal image.

### Image processing

**Fig 3:**
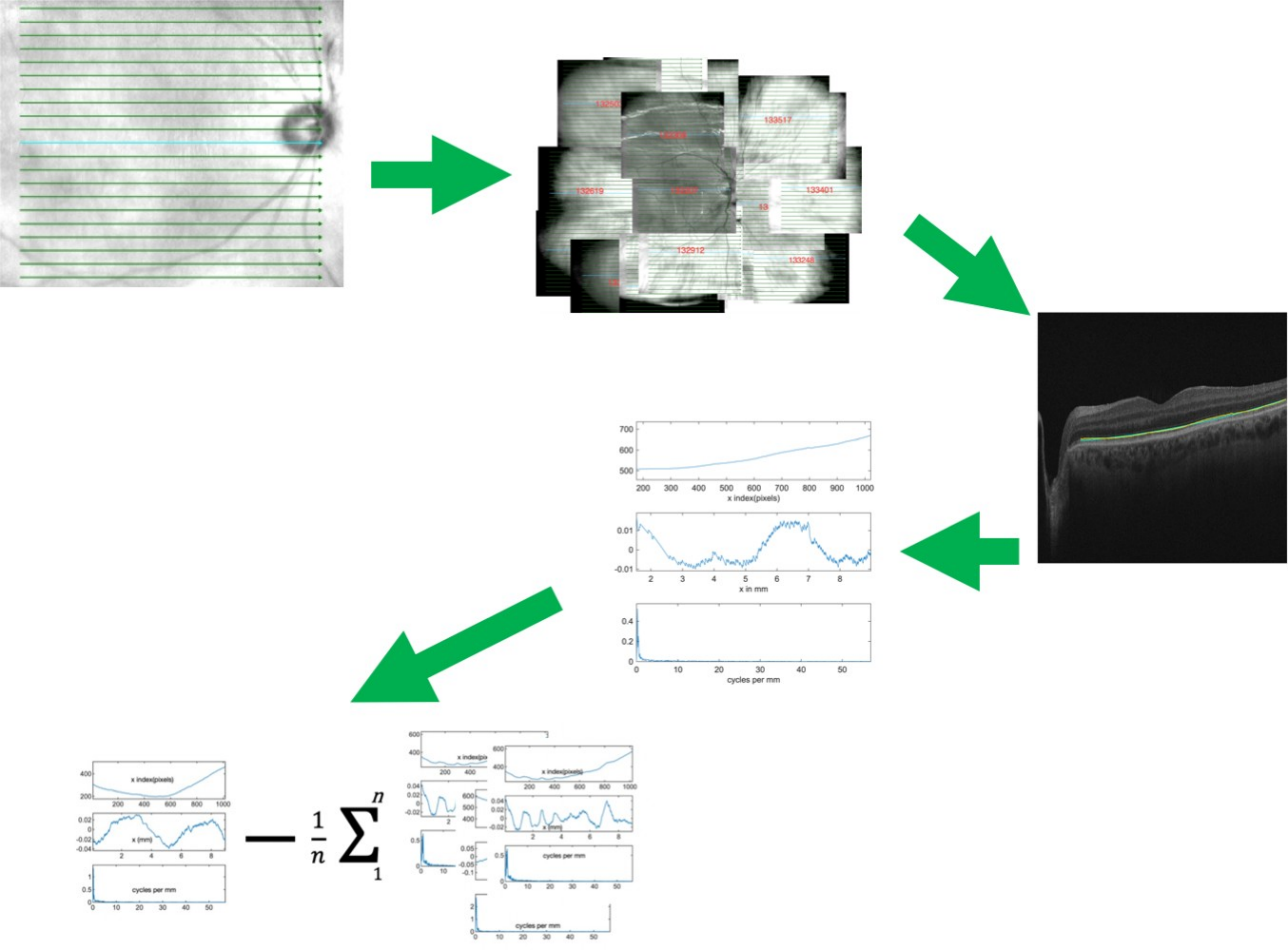
The process of extracting shape information from retinal OCT images. OCT cubes consisting of 21 parallel B scans 9 mm long and spaced out by 0.4 mm were taken of multiple locations within the eye. The retinal contour, taken as the path the retinal pigment epithelium takes across each individual B scan was identified. This was described by a best fit second order polynomial curve, and the Fourier transform of the residual after the best fit curve was subtracted. These were compared to the average value of 80% of the B scans within the group.

Raw IMG data files were exported from the OCT device (Fig 3). These were converted to tiff file format, and in each B scan the retinal shape was captured using the Livewire plugin [20] for ImageJ.[21] The retinal shape was represented by the retinal pigment epithelial line or outer highly reflective band in all but the posterior (macular) scans. Macular shape information was taken from the ellipsoid line, as this high intensity line was more reliably tracked with Livewire, and is parallel to (and therefore has the same shape as) the retinal pigment epithelial line in the images acquired. Text files of the retinal coordinate information were imported into MATLAB (The MathWorks, Inc., Natick, MA).[22]

### Measurement of retinal shape

**Fig 4:**
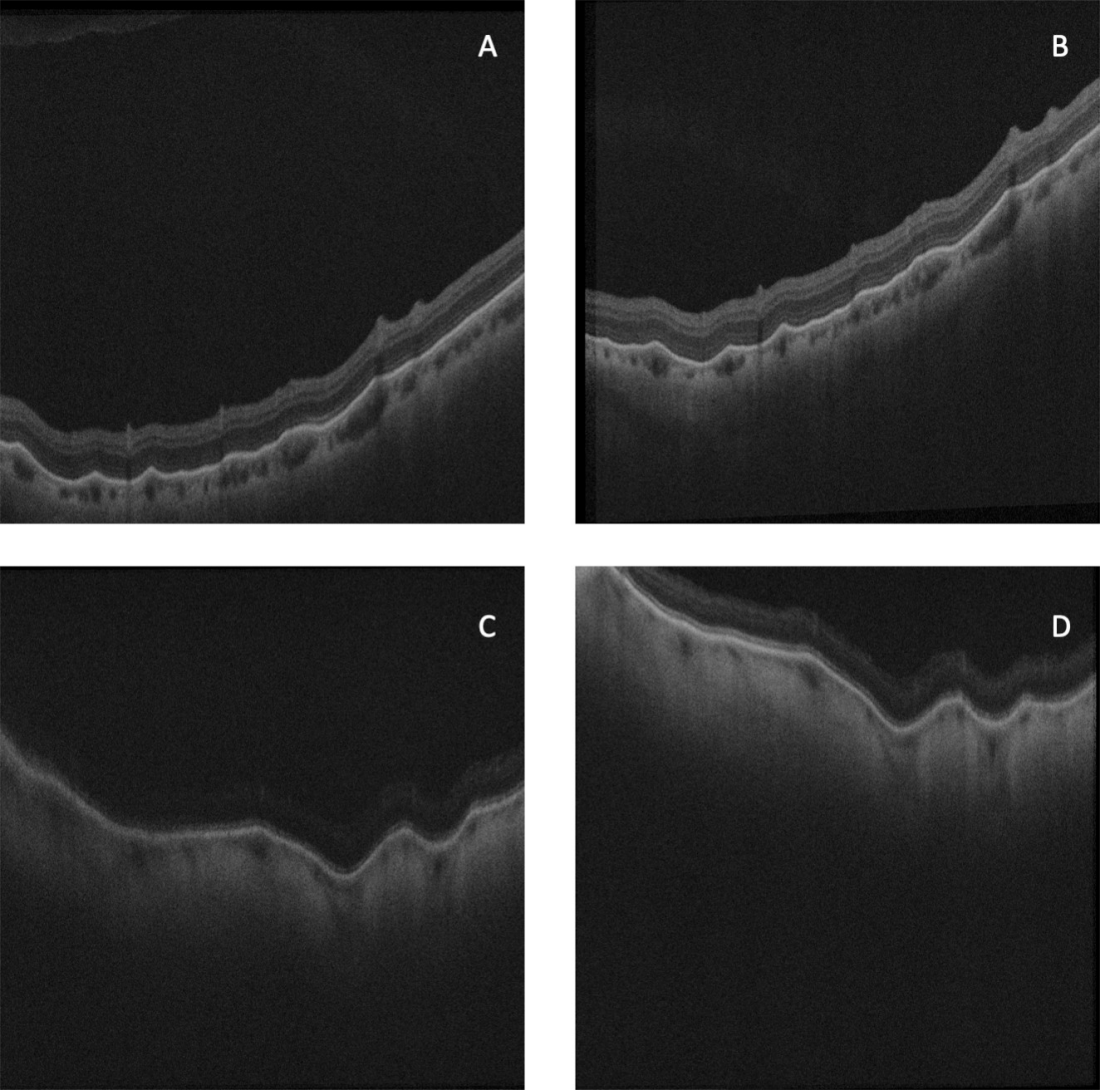
Repeat B scans from inferior retinal regions of two separate eyes. Scans A and B were from OCT cubes of the same region of one eye taken on separate days, and scans C and D scans from cubes taken from the same region of a different eye, also on separate days. Despite differing position within the B scan window, retinal shape features in the first scan are seen repeated in the second scan in each eye. These images are presented isotropically (pixel size the same horizontally and vertically). In the eye, these retinal sections would be five times wider than their height.

Fig 4 illustrates peripheral retinal features that have been imaged at two separate sittings. There is a good correspondence between the images despite variation in position within the scan window–there are rotational and translational differences between images of similar retinal areas. Each OCT cube is in its own coordinate system, and can be rotated and translated compared to other images of the same or different eyes.[23] Orientation independent information on shape is required to compare images of the same area of retina taken at differing orientation. Analysis of shape depends on features that are independent of rotation, translation or scaling.

The retinal shape data consisted of two columns, representing the x (lateral) and z (axial) coordinates for each pixel representing the retinal shape in the B scan. This coordinate information was converted from pixels to physical length in millimetres. Analysis was performed with script written in MATLAB for this study.

For duplicate values of x, a single z value was attached to this x value equal to the mean of the z values recorded for the duplicate x values. Gaps in the sequence of x values were filled by linear interpolation. The best-fit quadratic was generated by the polyfit function in MATLAB, and subtracted from the retinal line (Fig 1). This was done to avoid contamination of the Fourier transform (aliasing) by mismatches at the endpoints of the signal. If the signal (length of the x vector) was less than 1024 points long, then the signal was padded by zeros to equal 1024. If the signal was more than 1024 points long, the ends were cut to limit the length to 1024. The discrete Fourier transform of the z values was computed and since z was a signal of length 1024, the modulus for the Fourier transform was defined on 512 evenly spaced frequency values in the range 0 to Ny (Nyquist frequency). The moduli of the Fourier transform of the 30 lowest frequency bins constituted the output vector representing the shape of the B scan. Higher frequency information was discarded as noise. The moduli for each frequency bin were corrected for the length of the signal (the adjusted length of the retina in the B scan) to allow comparison of images of different size, as not all retinal images in peripheral retina extend from one side of the B scan to the other (Fig 2).

To account for shape information within the best-fit quadratic that was subtracted from the retinal shape prior to Fourier transformation, the curvature at the vertex of the best-fit curve was recorded for each B scan (hereafter K). Curvature was derived geometrically rather than optically, and was used to differentiate OCT shape from other measurements in vision science that use radius of curvature. It has the advantage that its magnitude increases as the curve becomes more acute. As the optics of longer eyes affect the measured curvature,[16] correction for axial length induced error was performed by converting curvature values to radius of curvature, deducting the appropriate correction, and recalculating K. Correction factors are reported for eyes with integer axial length 21–28 mm, so within and beyond this range (nine eyes had an axial length greater than 28 mm) the actual correction value was calculated from a second order polynomial fit to the available data. The median K for a single cube reflected the typical curvature within the volume of that cube. The interquartile range of K for a single cube was the interquartile range of the curvature of the B scans within that cube. It represented the degree to which curvature changes across the area of the cube from top to bottom, and not the spread of observations from a single sample. For an entire eye, the interquartile range K represented the range of values within the eye. Some B scans had negative curvature (convex into the eye), for instance at the edge of staphyloma.

**Fig 5:**
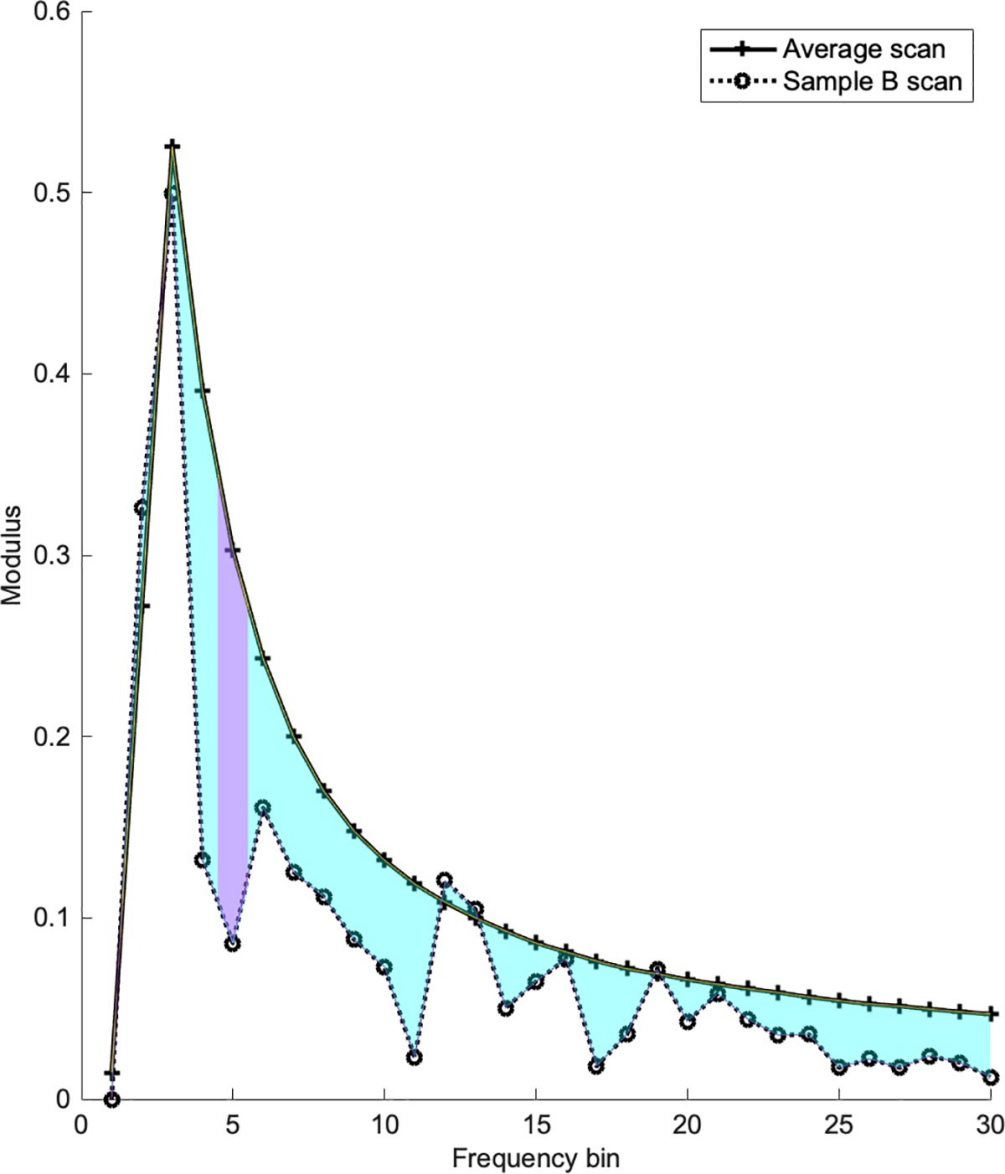
Illustration of features sumdiff and MaxE. The average frequency bin value for all B scans is illustrated by the solid line. The coloured shaded areas (cyan and lilac) represent sumdiff for the sample B scan (dotted line). MaxE is the bin value with the largest difference from the average, and represented by the lilac shaded area.

The difference between the retinal shape and its best-fit quadratic curve was represented by three variables derived from the Fourier transform. The first (labelled sumdiff) was the sum of the difference between the 30 lowest frequency bin moduli of a scan and the average normal values of the same frequency bins (derived below). The second variable was the largest single frequency bin difference in a B scan compared to average bin values (MaxE). The third was the root mean square of the difference between the scan bin moduli and the average normal bin values, named rmse. These variables reflected the difference of any B scan from the average B scan in its control group. More irregular retinal shapes that correspond poorly to a quadratic (parabolic) curve had greater sumdiff, MaxE, and rmse. The interquartile range of sumdiff, MaxE and K was used to represent the spread of values within each eye or region. A description of these variables is presented in Table 1, and Fig 5.

**Table 1:** Shape descriptors.

Variable	Description
sumdiff	sum of the difference between scan bin moduli (value) and the average bin moduli, for the 30 lowest frequency bins. Sumdiff equates to area between the best fit curve and the RPE.
MaxE	Greatest single bin difference between a B scan modulus and its corresponding average bin modulus value.
rmse	root mean square of the difference between sample B scan bin moduli and the average bin moduli.
K	curvature at the vertex of the best-fit quadratic curve to the retinal contour.

A description of the summary variables used for shape analysis. Sumdiff, MaxE, and rmse are derived from the Fourier transformation of the residual after the best fit quadratic curve is subtracted from the retinal contour.

The average bin value for comparison was determined from 80% of the entire cohort, by dividing into five groups. Each group was balanced by sorting the eyes by axial length, then randomly distributing each consecutive five eyes into the five groups. Numbers in each group were non-equal when the sample was not divisible equally by five, when the remaining ‘n’ samples were randomly placed into ‘n’ groups. The eyes in each group were compared to the average value derived from the other four groups combined. For regional analyses B scans were compared to an average value calculated only from B scans from the same region. For all-of-eye features B scans from all regions were used to create the average value.

### Statistical methods

All-of-eye and regional median and interquartile range of features sumdiff, MaxE, rmse, and K were correlated to axial length by Spearman’s rank correlation with 95% confidence intervals calculated with Fisher’s z-transformation. Bonferroni-Holm correction for multiple comparisons was performed with alpha set to 0.05. Eyes that were scanned twice were compared by the Wilcoxon signed rank sum test.

## Results

**Fig 6:**
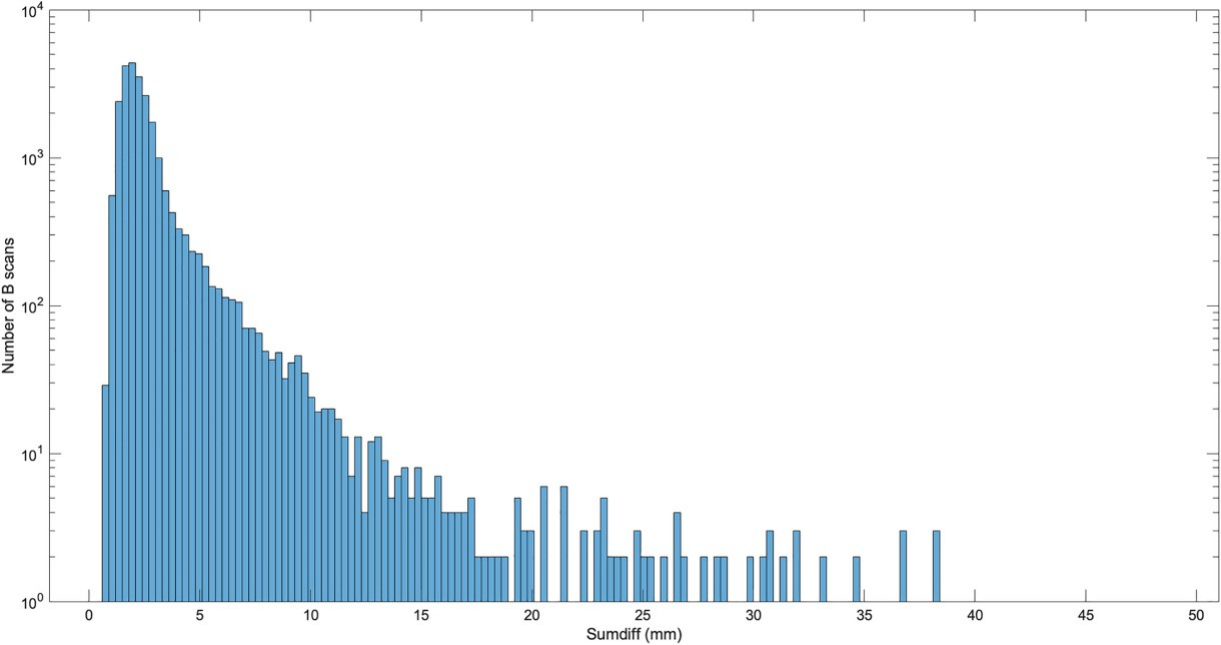
Distribution of sumdiff values, all Bscans. The number of B scans for each value of sumdiff are shown. Note the y-axis is a logarithmic scale: 93% of B scans have sumdiff less than 5 mm. Median sumdiff is 2.14 mm (interquartile range 1.04 mm).

**Fig 7:**
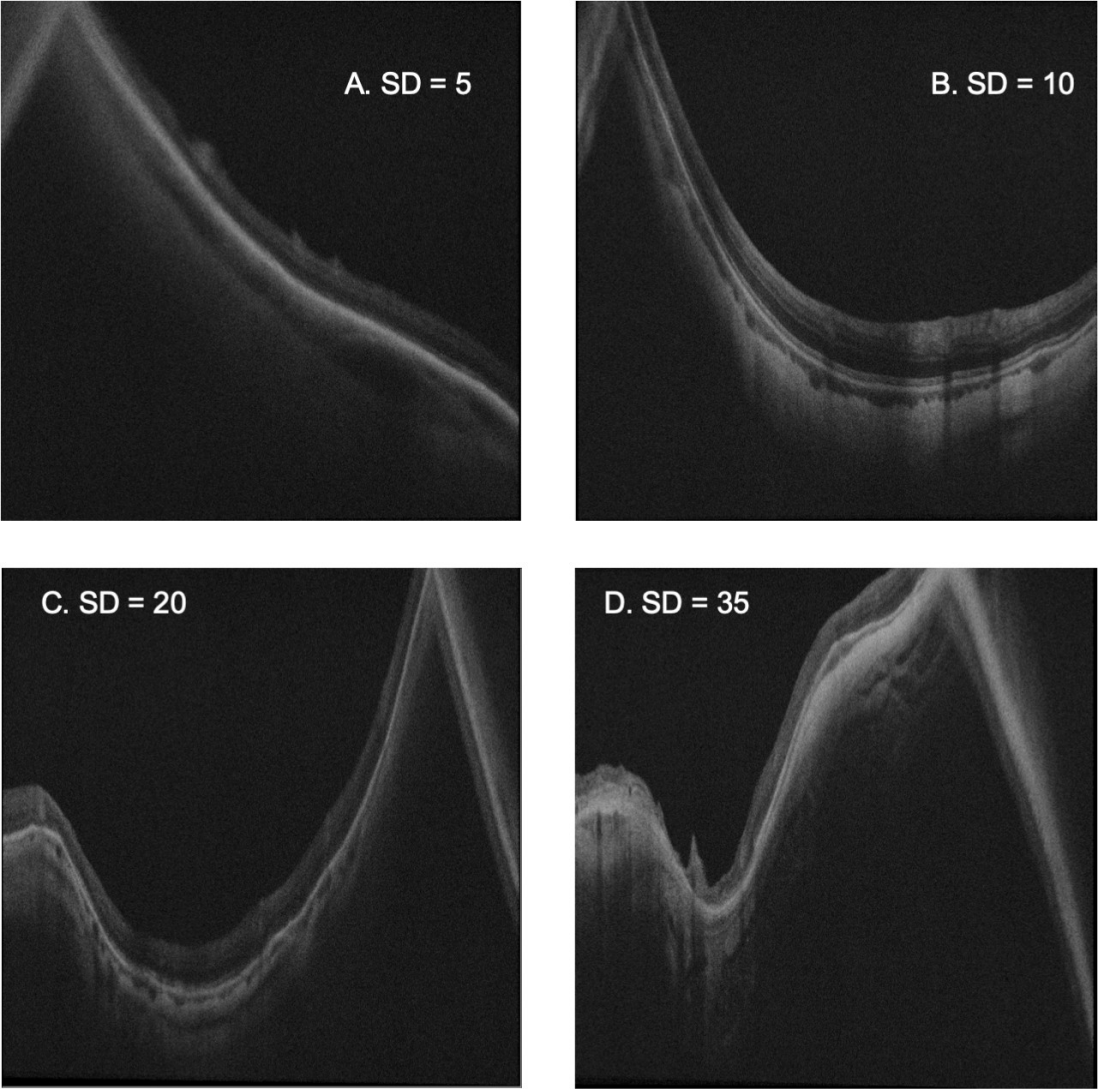
Sample B scans by sumdiff value. B scans with sumdiff 5 mm (A), 10 mm (B), 20 mm (C), and 35 mm (D) are presented. All feature values are corrected for the retinal length in the B scan, to allow comparison between images. Retinal mirror artefacts (to the left side in images A & B, and the right side of C & D) are ignored.

The distribution of magnitude of sumdiff is illustrated by a histogram in Fig 6. B scans giving examples from sumdiff magnitudes 5, 10, 20, and 35 mm are presented in Fig 7.

### Correlation between all-of-eye shape variables and axial length

**Fig 8:**
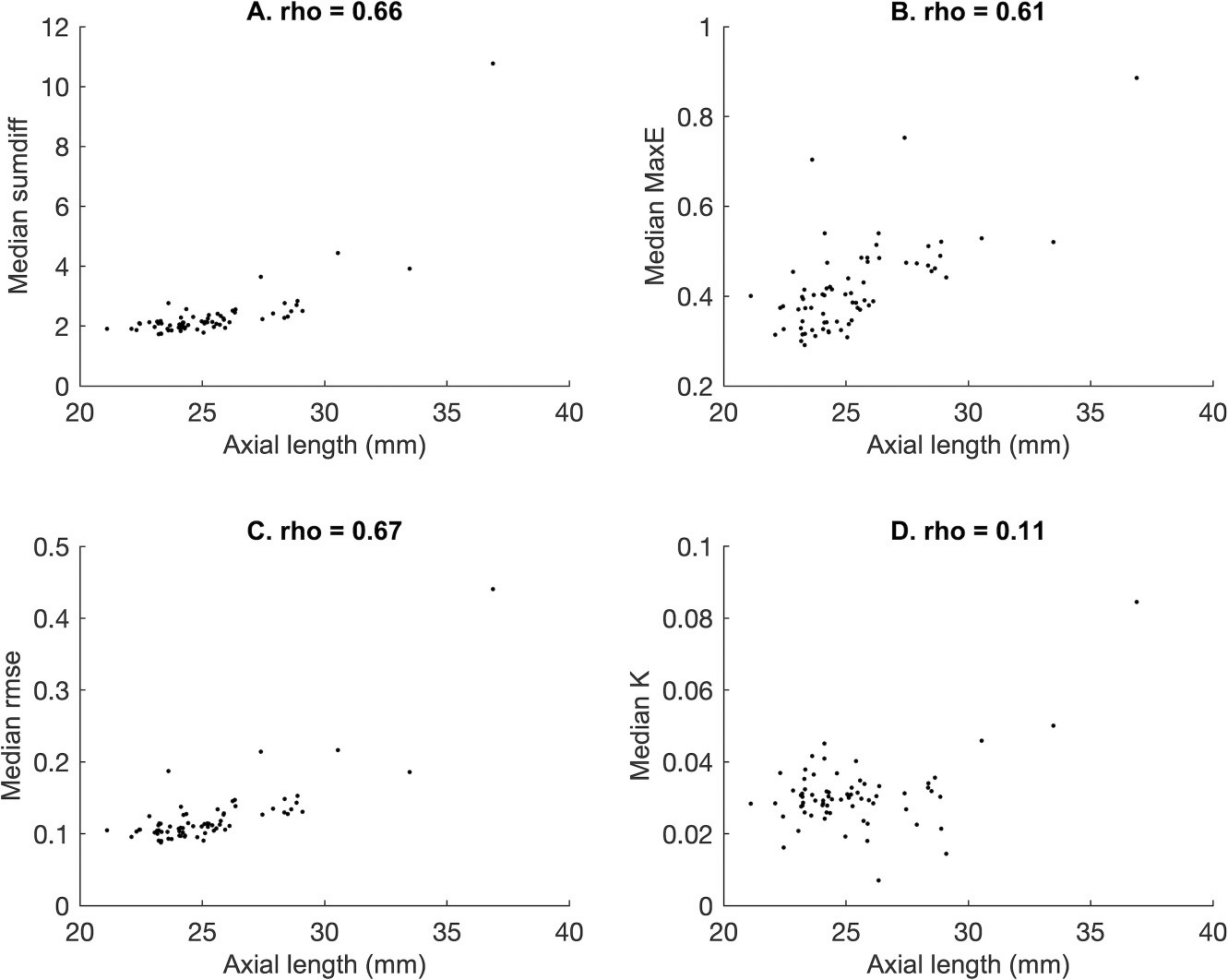
The correlation between retinal shape metrics and axial length. Scatter plots of whole-of-eye features median sumdiff (A), median MaxE (B), median rmse (C), and median K (D) against axial length. Spearman’s rank ***ρ*** is given for the correlation in each plot, and was significant for A-C (see Table 2).

Bonferroni-Holm correction for multiple comparisons required p < 0.00046 with α = 0.05 for significance. After correction, there was significant positive correlation between axial length and all-of-eye median sumdiff (***ρ*** = 0.66), MaxE (***ρ*** = 0.61), and rmse (***ρ*** = 0.67). No correlation was seen between eye median K and axial length (***ρ*** = 0.11). Interquartile range sumdiff (***ρ*** = 0.60), interquartile range rmse (***ρ*** = 0.48), and interquartile range K (***ρ*** = 0.61) also correlated with axial length. Full results for all-of-eye variables are shown in Tables 2 and 3, and Fig 8. Correlation was repeated with the largest eye (axial length 36.88 mm) removed in case this eye was skewing the results, with almost identical results (see Supplementary S1 Table).

**Table 2:** Correlation between median shape metrics and axial length.

Correlation *ρ*	median	median	median	median
	Sumdiff (95% CI)	MaxE (95% CI)	rmse (95% CI)	K (95% CI)
all of eye	**0.66 (0.49–0.77)**	**0.61 (0.46–0.75)**	**0.67 (0.52–0.78)**	0.11 (-0.14–0.32)
Macula	**0.52 (0.34–0.68)**	**0.50 (0.32–0.67)**	**0.50 (0.33–0.67)**	0.32 (0.10–0.53)
Posterior superior	**0.47 (0.24–0.62)**	**0.46 (0.20–0.59)**	**0.49 (0.25–0.63)**	0.16 (-0.16–0.31)
Anterior superior	0.42 (0.19–0.60)	0.35 (0.11–0.55)	0.41 (0.19–0.60)	0.17 (-0.16–0.33)
Posterior ST	0.33 (0.07–0.52)	0.32 (0.08–0.53)	0.35 (0.11–0.55)	-0.22 (-0.45–0.02)
Anterior ST	0.08 (-0.15–0.34)	0.11 (-0.15–0.34)	0.08 (-0.12–0.37)	-0.02 (-0.26–0.23)
Posterior temporal	**0.44 (0.21–0.61)**	0.34 (0.17–0.59)	0.38 (0.22–0.62)	-0.18 (-0.37–0.12)
Anterior temporal	0.09 (-0.16–0.33)	0.31 (0.10–0.54)	0.21 (-0.01–0.46)	0.11 (-0.18–0.32)
Posterior IT	**0.48 (0.24–0.64)**	0.37 (0.14–0.57)	0.42 (0.19–0.61)	-0.38 (-0.50–-0.04)
Anterior IT	0.28 (-0.03–0.44)	0.29 (0.06–0.51)	0.34 (0.04–0.49)	-0.30 (-0.49–-0.03)
Posterior inferior	**0.56 (0.38–0.70)**	**0.49 (0.30–0.66)**	**0.49 (0.30–0.66)**	0.32 (0.15–0.56)
Anterior inferior	0.20 (-0.04–0.42)	0.10 (-0.14–0.33)	0.17 (-0.09–0.38)	0.03 (-0.21–0.27)
Posterior IN	**0.47 (0.27–0.65)**	0.41 (0.19–0.59)	**0.45 (0.25–0.63)**	0.13 (-0.11–0.36)
Anterior IN	0.35 (0.13–0.56)	0.29 (0.09–0.53)	0.29 (0.01–0.47)	-0.15 (-0.44–0.02)
Posterior nasal	0.35 (0.10–0.53)	0.30 (0.06–0.51)	0.35 (0.09–0.53)	0.21 (-0.09–0.39)
Anterior nasal	0.14 (-0.08–0.40)	0.05 (-0.22–0.27)	0.14 (-0.13–0.36)	-0.09 (-0.35–0.13)
Posterior SN	0.41 (0.18–0.58)	**0.44 (0.21–0.60)**	0.41 (0.18–0.58)	0.11 (-0.18–0.30)
Anterior SN	**0.61 (0.44–0.75)**	**0.47 (0.31–0.68)**	**0.59 (0.44–0.75)**	-0.17 (-0.43–0.05)

Spearman’s rank correlation ***ρ*** between axial length and median sumdiff, MaxE, rmse, and K. See Table 3 footnotes for full description.

**Table 3:** Correlation between within-group interquartile range of shape metrics and axial length.

Correlation *ρ*	IQR	IQR	IQR	IQR
	sumdiff (95% CI)	MaxE (95% CI)	rmse (95% CI)	K (95% CI)
all of eye	**0.60 (0.41–0.72)**	0.06 (-0.18–0.29)	**0.48 (0.28–0.64)**	**0.61 (0.44–0.74)**
Macula	**0.47 (0.26–0.63)**	**0.53 (0.34–0.68)**	**0.52 (0.26–0.63)**	**0.44 (0.26–0.63)**
Posterior superior	0.25 (0.01–0.46)	0.24 (0.03–0.47)	0.26 (0.03–0.47)	0.20 (-0.01–0.44)
Anterior superior	0.33 (0.11–0.55)	0.34 (0.17–0.59)	0.37 (0.14–0.57)	0.11 (-0.20–0.29)
Posterior ST	0.16 (-0.09–0.40)	0.11 (-0.20–0.30)	0.13 (-0.15–0.34)	0.34 (0.09–0.54)
Anterior ST	0.00 (-0.20–0.29)	-0.06 (-0.22–0.27)	-0.06 (-0.24–0.25)	0.07 (-0.20–0.29)
Posterior temporal	**0.44 (0.22–0.62)**	0.13 (-0.11–0.38)	0.28 (0.09–0.53)	**0.47 (0.24–0.64)**
Anterior temporal	0.33 (0.09–0.53)	0.12 (-0.27–0.22)	0.21 (-0.06–0.42)	0.23 (0.00–0.47)
Posterior IT	0.41 (0.19–0.60)	0.10 (-0.23–0.27)	0.20 (-0.09–0.40)	0.31 (0.03–0.49)
Anterior IT	0.29 (0.05–0.51)	0.16 (-0.10–0.38)	0.28 (0.02–0.48)	**0.47 (0.26–0.65)**
Posterior inferior	**0.48 (0.27–0.64)**	0.32 (0.08–0.51)	0.29 (0.08–0.51)	0.35 (0.12–0.54)
Anterior inferior	0.04 (-0.20–0.28)	-0.16 (-0.34–0.14)	-0.12 (-0.33–0.14)	0.31 (0.05–0.50)
Posterior IN	0.37 (0.13–0.55)	0.08 (-0.18–0.30)	0.26 (-0.01–0.45)	**0.45 (0.26–0.64)**
Anterior IN	0.29 (0.05–0.49)	0.08 (-0.19–0.29)	0.18 (-0.05–0.42)	0.20 (-0.07–0.40)
Posterior nasal	0.27 (0.01–0.46)	0.14 (-0.17–0.32)	0.20 (-0.08–0.39)	**0.57 (0.36–0.70)**
Anterior nasal	0.39 (0.15–0.57)	-0.06 (-0.27–0.22)	0.16 (-0.12–0.36)	**0.45 (0.23–0.62)**
Posterior SN	0.25 (0.00–0.45)	0.15 (-0.13–0.34)	0.20 (-0.04–0.42)	**0.58 (0.40–0.72)**
Anterior SN	**0.52 (0.32–0.68)**	0.04 (-0.19–0.30)	0.37 (0.15–0.57)	0.43 (0.24–0.64)

Spearman’s rank correlation ***ρ*** between axial length and median (Table 2) and interquartile range (Table 3) sumdiff, MaxE, rmse, and K. Values significant after Bonferroni-Holm correction for multiple comparison are highlighted in bold, and for all these values p < 0.001. Correlation between irregularity (median sumdiff, MaxE, and rmse) and axial length was greater in macula and posterior extra-macular cubes than in anterior extra-macular cubes. Range of curvature (IQR K) correlated with axial length in most nasal regions. IQR = interquartile range, ST = supero-temporal, IT = infero-temporal, IN = infero-nasal, SN = supero-nasal.

### Correlation between regional measures and axial length

Bonferroni-Holm correction for multiple comparison with α = 0.05 required p < 0.00046 for significance with regional and all-of-eye correlations combined, and 36 values met this criterion (in bold type in Tables 2 and 3). Correlation between regional irregularity and axial length was strongest in macula and posterior extra-macular cubes as measured by median sumdiff, MaxE, and rmse. Interquartile range of curvature correlated with axial length in most nasal regions (Table 3).

**Fig 9:**
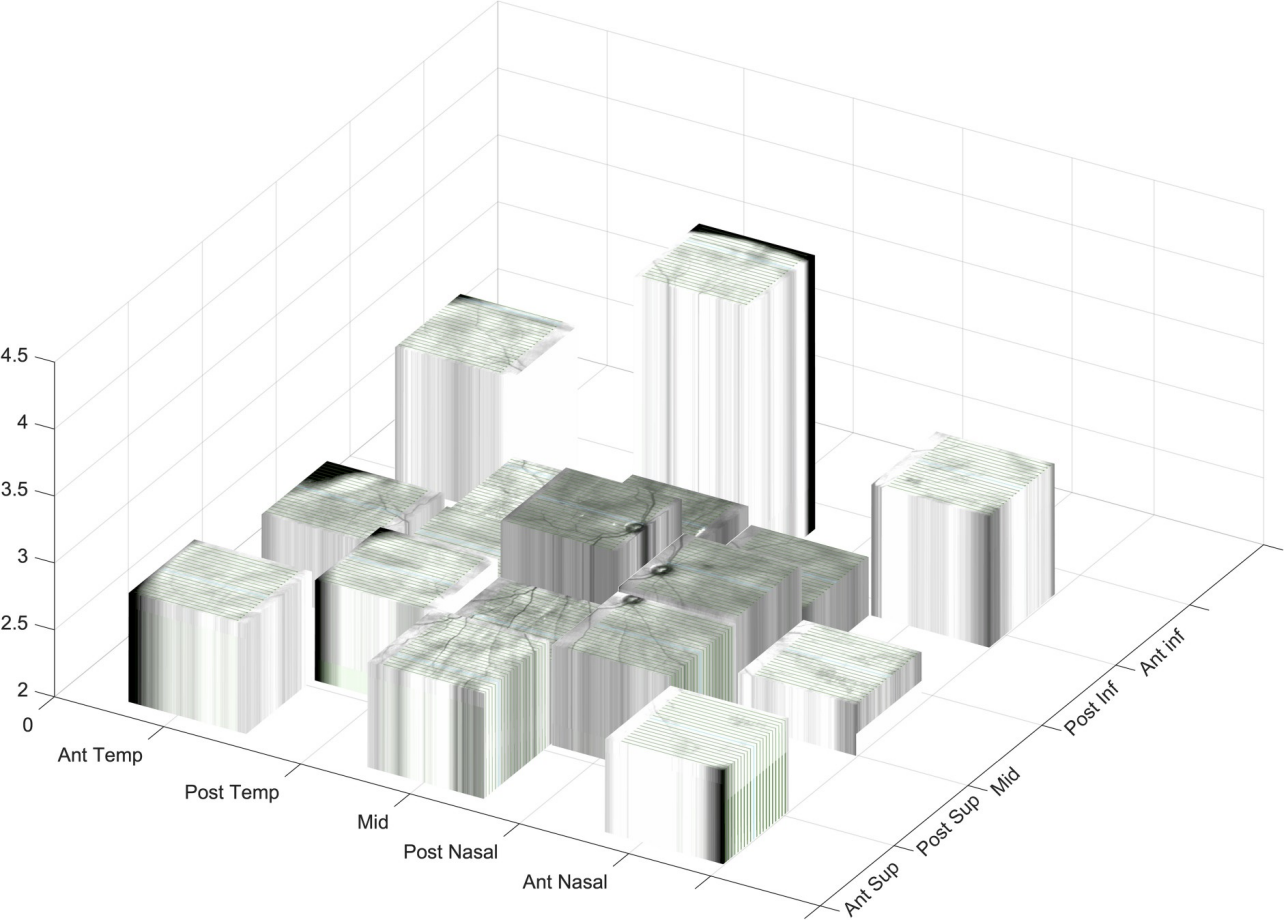
Retinal OCT shape irregularity in different regions of the eye. Graphical representation of the magnitude of irregularity (the sum of the first 30 frequency bins of the Fourier transform of B scan shape) in different regions of the eye, viewed from a viewpoint supero-nasal to the eye (looking down on a left eye from a point above the medial end of the eyebrow). The vertical axis scale is in mm, so the taller the column the greater the irregularity (values are reported in column 1 of Table 4). The anterior inferior and macula regions are the most irregular locations.

The magnitude of irregularity in different regions is illustrated in Fig 9. The anterior inferior regions had the highest values, followed by the macula. These regions had the most irregular retinal contour that corresponded least with a best-fit quadratic curve, regardless of axial length. Regional median sumdiff, MaxE, rmse, and K values are presented in Table 4. The macula cube’s increasing irregularity in larger eyes was reflected by the higher sumdiff, MaxE, and third highest median sum of frequency bins.

**Table 4:** Shape description of retinal regions.

Region	Median sum bins	Median sumdiff	Median MaxE	Median rmse	Median K	IQR sumdiff	IQR MaxE	IQR rmse	IQR K
Macula	2.992	2.445	0.515	0.139	0.026	1.511	0.368	0.087	0.020
Posterior superior	2.624	1.838	0.387	0.108	0.026	1.043	0.275	0.068	0.018
Anterior superior	2.814	1.910	0.417	0.114	0.018	1.160	0.289	0.077	0.025
posterior supero-temporal	2.730	1.813	0.400	0.109	0.028	0.927	0.284	0.064	0.023
Anterior supero-temporal	2.752	2.235	0.327	0.109	0.036	1.050	0.168	0.062	0.027
posterior temporal	2.453	1.681	0.341	0.093	0.032	0.886	0.242	0.056	0.021
Anterior temporal	2.520	2.018	0.257	0.090	0.039	0.819	0.094	0.039	0.028
posterior infero-temporal	2.433	1.671	0.349	0.095	0.031	0.803	0.207	0.050	0.025
Anterior infero-temporal	3.015	2.170	0.327	0.107	0.031	0.884	0.159	0.056	0.032
posterior inferior	2.615	1.906	0.404	0.109	0.032	1.131	0.297	0.078	0.019
Anterior inferior	4.066	3.017	0.727	0.188	0.031	1.814	0.387	0.117	0.026
posterior infero-nasal	2.434	1.766	0.337	0.096	0.034	0.819	0.211	0.052	0.018
Anterior infero-nasal	2.969	2.310	0.402	0.121	0.029	1.200	0.169	0.072	0.024
posterior nasal	2.795	1.989	0.382	0.108	0.036	0.958	0.263	0.063	0.021
Anterior nasal	2.381	1.760	0.211	0.079	0.028	0.763	0.136	0.041	0.025
posterior supero-nasal	2.736	1.962	0.401	0.109	0.027	1.027	0.280	0.064	0.019
Anterior supero-nasal	2.676	2.078	0.372	0.108	0.026	1.028	0.175	0.062	0.025

Eight eyes were scanned twice on separate days with axial length ranging from 22.91–27.21 mm, and age range 28–73 years, and compared by Wilcoxon signed rank sum test of maximum, minimum, median, and interquartile range of regional descriptors sumdiff, MaxE, rmse, and K. No significant difference was detectable between repeat scans (p values 0.10–0.94).

Regional shape descriptor (defined in Table 1) values. The first column is the sum of the 30 lowest frequency bin median moduli for each region. The regions with the largest values (the macula and anterior inferior regions) were more irregular than regions with smaller values. Descriptors sumdiff, MaxE, and rmse describe the variation in irregularity seen in B scans within a region, compared to the average B scan irregularity within the same region. Median sumdiff = region median absolute difference from the average region value. Median MaxE = median largest single frequency bin difference from the region average. rmse = root mean square difference between each B scan and the average bin value. K = curvature at the vertex of the best-fit curve to the retinal shape. Units are mm for sum bins, sumdiff, MaxE, and rmse, and units are mm^-1^ for K.

## Discussion

The ability to quantify OCT retinal shape allows comparison of multiple scans within and between eyes. Median and interquartile range are a conservative choice of metrics to represent retinal shape, which limit the impact of outlying values on the analysis. The shape descriptors derived from the Fourier transform and best-fit curvature are unaffected by position and orientation of the retinal image within the B scan. Some distortions of retinal contour occur during OCT image capture, most significantly due to axial length, and correction of curvature for axial length induced artefact was performed.[16,24] Optical distortions from anterior chamber structures may alter retinal topography,[25] but both tilt of the eye and lateral decentration have little effect on retinal geometry in OCT.[16,26] The speed of A scan acquisition and the use of composite B scans preserves real information on retinal shape within the OCT image. Just as the pattern on an embossed leather belt is recognisably the same whether it is held straight or flexed, so the information within the Fourier transform of retinal irregularity reflects real retinal features.

Participant eyes were included in this study if they were found to have no abnormality on examination (other than the myopic retinal changes described in the Methods), as the purpose was to explore and describe retinal shape features in healthy eyes. This analysis does not identify the cause of retinal irregularity. The progression in irregularity in retinal shape was large to be entirely explained by myopic choroidal thinning. Some of the irregularity was likely to be related to staphylomata, but the identification of greater irregularity in inferior retinal regions, irrespective of axial length, was a novel finding. While it may partly relate to alterations in choroidal thickness, the irregular contour seen in dome-shaped maculopathy has been reported to be due to variations in scleral thickness leading to alterations in scleral rigidity.[27] Spectral domain OCT does not have sufficient resolution at depth to discriminate between these possibilities, particularly in mid-peripheral retina where signal strength is reduced. Imaging with swept source OCT may clarify this further. This method provides a quantitative description of the deformation of the retinal contour that arises from overall eye size. Further work is planned to explore whether there is any correlation between retinal contour irregularity and eye disease, in which awareness of the link between axial length and irregularity will have to be incorporated.

A quadratic curve was chosen for the best-fit curve due to its simplicity and the utility of the vertex curvature remaining unaffected by changes in orientation. Retinal areas sampled by OCT may correspond better to an elliptical or hyperbolic form, but the general conic equations for these are orientation specific. Calculation of radii of curvature for elliptical or hyperbolic curves, while orientation independent, is a poorly constrained problem as all the information on the retinal shape is confined to a short arc (the retinal OCT image). Minor changes in retinal position lead to widely differing solutions for the radii, rendering the information unreliable.

When comparing cubes by region, classification of cube location was based on examination of the scanning laser ophthalmoscope image taken simultaneously with OCT image capture. Retinal shape irregularities remain unchanged when re-imaged weeks apart (Fig 4). For the reliability test with repeat eye scans, no attempt was made to exactly match the cube location, rather each cube was classified by its general region. It was reassuring that no significant difference was seen on retest, implying measurements were robust to small translations of cube position. Re-scanning eyes did not demonstrate any significant difference between repeat examinations, but it should be acknowledged that statistical tests are methods of assessing dissimilarity, and not designed to confirm that two samples (in this case re-tests of the same eye) have the same identity.

The advantage of Fourier transformation for residual analysis is that the same shape features in a different position in two scans have the same Fourier domain magnitude irrespective of where in the scan the features are located. It also allows examination of the composition of irregularity, which here is confined to the greatest bin variation between scans (MaxE). Best-fit curve vertex curvature also remains the same irrespective of orientation. Despite the high likelihood of incomplete overlap between cubes of re-scanned regions, no significant difference was found between two separate examinations of eyes performed on different days, supporting the reliability of these measures.

Scans taken of more peripheral areas, particularly those away from the horizontal and vertical meridians, exhibited a reduced angle between the scan axis and the retinal surface. This might have influenced the fit of the retinal image to a parabola (the shape of a quadratic curve), but all regional cube comparisons were to cubes within the same region and will have a similar orientation. Eyes differ in size, and it is not possible to scale or match the sample volume in one eye to that of another, but the use of comparable areas as a reference for each scan should limit the impact of the changing acquisition angle on the results.

The measurements presented above confirm impressions gathered from visual inspection of B scans in different parts of the eye. Irregularity, measured by Fourier analysis of the residual, increased with axial length at and around the macula. Interquartile range of curvature correlated with axial length at the macula and in most nasal regions, so these areas had a wider range of curvatures as axial length increased. The shape metrics of the anterior inferior and infero-nasal regions had no correlation to axial length. The inferior regions had the most irregular retinal contour on OCT irrespective of axial length (the images in Fig 4 are from inferior retinal regions). It may be that the heterogeneity of shape in these regions masks any correlation between axial length and shape.

The correlation between axial length and OCT retinal shape variation was expected from clinical and MRI observation of globe shape, but has not previously been described with the resolution of the OCT. In myopic eyes, the macula can be abnormal in shape with highly curved concave sections from staphylomata. Posterior non-macular cubes in myopic eyes image the margins of staphylomata and have negative K (convex inwards), and OCT cubes anterior to this often display a straight retinal contour. These factors reduce median curvature values in myopic eyes while increasing the range of curvature values and explain why median K does not correlate with axial length while interquartile range K does.

Measurement of variation in local curvature and the Fourier analysis of differences between OCT retinal contour and the best-fit curvature provide a measure of regional variation in shape. The correlation between shape features and axial length agrees with the known irregularity of the eye in increasing myopia on MRI. As the OCT has much greater resolution than MRI, retinal shape features reported here are so small that MRI images are unlikely to be able to confirm or refute the measurements taken with the OCT. Although retinal curvature analysis has been reported, this is the first report that systematically describes and quantifies the residual, or difference between measured curvature and actual retinal contour, and extends the analysis to the mid-peripheral retina. Local curvature cannot be extrapolated to represent the shape of the posterior segment as a whole. Retinal OCT shape irregularity increased with the axial length of the eye, and was greatest inferiorly. It can be used to describe myopic eye disease, and to examine retinal shape in other eye conditions.

## Supporting information

S1 File Study data file key. This describes the data held in ALIFb.mat.
(DOCX)

S2 File ALIFb.mat. MATLAB array with Fourier transform and axial length data for study eyes.
(MAT)

S1 Table Correlation between shape features and axial length, excluding largest eye. (DOCX)
