# Supplementary material for: The correlation between optical coherence tomography retinal shape irregularity and axial length
Source: PLoS One. 2019 Dec 30;14(12):e0227207. doi: 10.1371/journal.pone.0227207 (PMC6936846; doi:10.1371/journal.pone.0227207)
Supplement: S1 File — This describes the data held in ALIFb.mat. (DOCX) [file pone.0227207.s001.docx]

# Study data file key

The data used for this study are recorded in the MATLAB array ALIFb.mat. This is accessible using MATLAB.

Column 1 has the unique participant identifier. The first digit (‘1’ or ‘2’) denotes eye laterality (right and left respectively), other letters and digits reflect the order participants were recruited.

Column 2 cells contain, in order: axial length right eye (mm), axial length left eye (mm), age (years), and two trailing numbers not used for this study. For most eyes only the eye included in the study has axial length recorded, the fellow eye identified by ‘0’.

Column 5 holds the Fourier transform moduli. Within each cell, rows are for frequency bins (30), columns for each B scan (maximum of 21 in each HD21 cube), and one OCT cube per sheet (third dimension).

Column 6 holds FFT data taken from column 5. This is for all the cubes that could be categorised into a region and used for regional analysis. The identifiers for these cubes are in column 4, and the identifiers of all the cubes in an eye are in column 3.
