# Supplementary material for: The correlation between optical coherence tomography retinal shape irregularity and axial length
Source: PLoS One. 2019 Dec 30;14(12):e0227207. doi: 10.1371/journal.pone.0227207 (PMC6936846; doi:10.1371/journal.pone.0227207)
Supplement: S1 Table — (DOCX) [file pone.0227207.s003.docx]

**Supplementary table S3.** Correlation of shape features to axial length, largest eye in series excluded. Results significant after multiple comparison correction are in bold, and are very similar to Table 2.

| **Correlation *ρ*** | **median** | **median** | **median** | **median** | **IQR** | **IQR** | **IQR** | **IQR** |
| --- | --- | --- | --- | --- | --- | --- | --- | --- |
|  | **sumdiff** | **MaxE** | **rmse** | **K** | **sumdiff** | **MaxE** | **rmse** | **K** |
| all of eye | **0.671** | **0.616** | **0.673** | 0.073 | **0.584** | 0.088 | **0.467** | **0.603** |
| Macula | **0.551** | **0.554** | **0.539** | 0.291 | **0.534** | **0.543** | **0.570** | **0.418** |
| Posterior superior | **0.493** | **0.431** | **0.487** | 0.145 | 0.273 | 0.249 | 0.273 | 0.210 |
| Anterior superior | 0.416 | 0.334 | 0.408 | 0.167 | 0.329 | 0.358 | 0.358 | 0.104 |
| Posterior ST | 0.315 | 0.324 | 0.353 | -0.216 | 0.159 | 0.126 | 0.169 | 0.347 |
| Anterior ST | 0.075 | 0.161 | 0.097 | -0.018 | 0.003 | 0.006 | -0.046 | 0.082 |
| Posterior temporal | **0.444** | 0.377 | 0.399 | -0.164 | **0.480** | 0.195 | 0.373 | **0.473** |
| Anterior temporal | 0.081 | 0.357 | 0.241 | 0.100 | 0.331 | 0.110 | 0.220 | 0.223 |
| Posterior IT | **0.489** | 0.387 | 0.415 | -0.364 | 0.411 | 0.136 | 0.194 | 0.306 |
| Anterior IT | 0.269 | 0.342 | 0.323 | -0.285 | 0.371 | 0.130 | 0.257 | **0.489** |
| Posterior inferior | **0.570** | **0.506** | **0.526** | 0.315 | **0.498** | 0.301 | 0.305 | 0.353 |
| Anterior inferior | 0.217 | 0.090 | 0.159 | 0.015 | 0.029 | -0.155 | -0.119 | 0.319 |
| Posterior IN | **0.473** | **0.426** | **0.455** | 0.128 | 0.369 | 0.020 | 0.261 | **0.456** |
| Anterior IN | 0.361 | 0.312 | 0.282 | -0.167 | 0.316 | 0.098 | 0.214 | 0.210 |
| Posterior nasal | 0.353 | 0.295 | 0.351 | 0.201 | 0.279 | 0.135 | 0.231 | **0.559** |
| Anterior nasal | 0.133 | 0.035 | 0.112 | -0.089 | 0.384 | -0.076 | 0.151 | **0.454** |
| Posterior SN | 0.409 | **0.445** | **0.418** | 0.098 | 0.291 | 0.193 | 0.271 | **0.586** |
| Anterior SN | **0.619** | **0.470** | **0.586** | -0.171 | **0.514** | 0.027 | 0.367 | 0.425 |
